# Postpartum Psychosis Presenting With Catatonia: A Case Study

**DOI:** 10.1192/bjo.2026.11868

**Published:** 2026-06-30

**Authors:** Zoe Mulligan, Sarah Smyth, Oladapo Babatola

**Affiliations:** Bluestone Unit, Craigavon, United Kingdom

## Abstract

**Aims:**

Postpartum psychosis is an acute and severe psychiatric illness, often characterised as a ’psychiatric emergency’ lacking distinct classification in the DSM-5. This leads to poor understanding of the condition and ambiguous management protocols. 1-2 per 1000 females are affected and this change in maternal behaviour can be characterised by affective mood symptoms but may also present with catatonic features. This case study highlights the challenge of recognising and initiating urgent management in the setting of postpartum psychosis.

**Methods:**

Female admitted at 14 days postpartum under detention following significant concerns regarding non-communication, markedly reduced oral intake, poor sleep and rapidly deteriorating mental state. The patient suffered a similar episode after her first child. She initially displayed heightened anxiety and elated behaviour followed by mutism and flat affect. She was treated for endometritis due to elevated inflammatory markers and treated for a lower limb deep vein thrombosis. Treatment was then commenced for postpartum psychosis presenting with catatonia with lorazepam, olanzapine and mirtazapine, titrated up to effect.

**Results:**

This case study highlighted the effectiveness of a ’lorazepam challenge’ with marked improvements in mental state seen within 48 hours and discharge home soon after. The importance of antenatal screening for high-risk patients is evident here, with emphasis on a previous episode of postpartum psychosis being the strongest predictor of recurrence in subsequent pregnancies (50% recurrence risk), which went undetected due to a language barrier. Also highlighted was the need to rule out organic causes initially in the workup for a diagnosis of postpartum psychosis.

**Conclusion:**

Postpartum psychosis is a condition requiring urgent attention and intervention with need for a multi-disciplinary team-based approach. Mother and Baby Units are vital in the setting of a postpartum psychosis in order to maintain the maternal-infant bond. Ongoing follow-up with the perinatal mental health team is vital in order to support the patient and family, to offer counselling and to provide information regarding recurrence risks. This case study is evidence of the need for more distinct classification and national guidelines to ensure accurate treatment is initiated in a timely manner.

